# Tunable Fractal Morphogenesis in Reaction-Diffusion Crystallization: From Dendrites to Compact Aggregates

**DOI:** 10.1021/acsomega.5c03338

**Published:** 2025-08-05

**Authors:** Seungju Moon, Mazen Al-Ghoul

**Affiliations:** Department of Chemistry, 11238 American University of Beirut, P.O. Box 11-0236, Riad El-Solh, 1107 2020 Beirut, Lebanon

## Abstract

Fractal growth in reaction-diffusion frameworks (RDF) offers a powerful paradigm for understanding self-assembly in chemical and materials systems. However, its connection to diffusion-limited aggregation (DLA) remains underexplored. Here, we present the first quantitative demonstration of RDF-driven fractal crystallization of benzoic acid (BA), revealing a direct correlation among fractal dimension, diffusion rate, and gel-matrix chemistry. In gelatin-based systems, BA crystallizes into dendritic structures that conform to classical DLA behavior, with fractal dimensions converging toward ∼1.71 to 1.74 at high supersaturation. Complementary characterization by powder X-ray diffraction and scanning electron microscope confirms consistent crystal structure across growth zones, while systematic peak shifts indicate uniform tensile macrostrain embedded during rapid, diffusion-limited growth. In contrast, agar-based systems yield spherulitic morphologies, underscoring the critical influence of gel-network interactions on crystallization pathways. Monte Carlo simulations of DLA in a concentric geometry further demonstrate that experimental fractal dimension trends map directly onto variations in effective sticking coefficients, indicating that supersaturation gradients modulate particle adhesion in the diffusion-controlled regime. Moreover, reverse-phase diffusion experiments reveal that slower diffusion promotes branch thickening and reduced fractal dimensions. These findings establish RDF crystallization as a versatile platform for engineering fractal architectures, offering new strategies for hierarchical material design, biomimetic crystallization, and soft-matter self-assembly.

## Graphical abstract

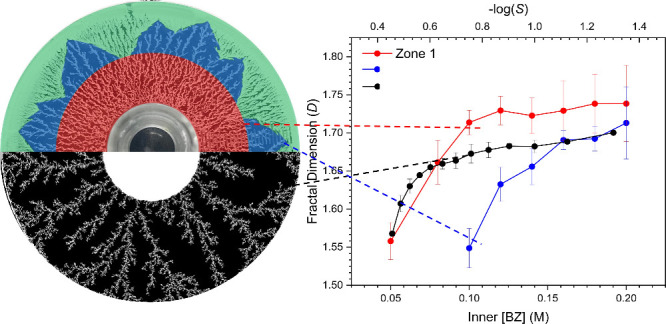

## Introduction

Fractal growth phenomena in reaction-diffusion frameworks (RDF) provide fundamental insights into self-assembly in chemical and material systems, yet their direct connection to diffusion-limited aggregation (DLA) remains underexplored. Fractals, characterized by their self-similar, scale-invariant morphology, emerge in various natural systems, from vascular networks to bacterial colonies.^1–6^ Among these, dendritic fractals exhibit stochastic aggregation behavior, closely modeled by DLA theory, which describes the growth of branched structures through random-walking particles adhering to an existing aggregate.^1^ DLA models typically yield fractal dimensions near 1.71,^7^ a universal characteristic of self-similar dendritic growth.^3,5^ However, while DLA principles have been widely studied in electrochemical deposition, colloidal aggregation, and biological systems, their application to molecular crystallization in a gel-confined reaction-diffusion environment remains largely unexplored.

Molecular crystallization under reaction-diffusion conditions introduces a new paradigm for studying DLA-like growth in soft-matter systems. Reaction-diffusion frameworks (RDF), when implemented using a gel medium, impose diffusional constraints on crystallization, suppressing convective flow and enabling local depletion zones to form around growing crystals.^8–12^ In such environments, crystal nucleation and growth become diffusion-controlled, allowing direct experimental access to fractal aggregation mechanisms previously confined to theoretical models. Also, the use of gel matrix has been shown to play a critical role in dendritic crystallization.^13–15^ Despite the potential significance of RDF in studying stochastic crystallization, its impact on molecular-scale fractal formation and crystallization kinetics remains underexplored. Benzoic acid (BA) serves as an ideal test system due to its well-characterized nucleation and precipitation behavior,^16–18^ making it a valuable model for elucidating fractal growth mechanisms in soft-matter crystallization. Thus, our system consists of H^+^ ions from the outer HCl solution (placed outside the gel medium) diffusing into the gel containing sodium benzoate, where the neutralization reaction between H^+^ ions and benzoate ions (BZ) leads to the rapid precipitation of benzoic acid.

Here, we present the first quantitative demonstration of RDF-driven fractal crystallization in a gel-confined system, revealing a direct correlation between fractal dimension, diffusion rate, and gel-matrix chemistry. Although dendritic morphologies have been previously observed in reaction-diffusion advection systems,^19^ such studies lacked quantitative analysis of the fractal dimension and its relations to parameters of classical DLA. By systematically varying gel networks (gelatin vs agar) and reactant concentrations, we demonstrate that BA crystallizes into dendritic fractals in gelatin, following classical DLA behavior with fractal dimensions converging toward 1.71–1.74. At the same time, spherulitic growth emerges in agar, underscoring the role of gel-matrix interactions in crystallization pathways. To establish a mechanistic connection between reaction-diffusion crystallization and stochastic aggregation models, we developed a Monte Carlo-based DLA simulation in a concentric geometry, confirming that experimental fractal dimension trends directly map onto variations in effective sticking coefficients. Additionally, reverse-phase diffusion experiments further validate that slower diffusion leads to branch thickening and reduced fractal dimensions, reinforcing the role of diffusion constraints in dictating fractal morphogenesis.

These findings establish RDF crystallization as a robust platform for engineering fractal architectures, bridging fundamental fractal growth models with real-world molecular self-assembly. By integrating reaction-diffusion principles, Monte Carlo modeling, and experimental crystallization, this study lays the foundation for future investigations into hierarchical material design, biomimetic crystallization, and self-organized pattern formation in controlled reaction-diffusion environments. Although not a full reaction-diffusion simulation, our model captures spatially modulated morphologies through a Monte Carlo-based DLA framework, linking experimental fractal dimensions to simulation-derived sticking coefficients.

## Experimental Section

### Materials

Sodium benzoate (C_6_H_5_COONa) and hydrochloric acid (HCl) were purchased from J.T. Baker and Merck, respectively, while agar (BD Bacto Agar) and gelatin (BD Difco Gelatin) were purchased from BD Biosciences.

### Preparation of Reaction-Diffusion Systems

The inner gel phase was prepared by dissolving gelatin (8% w/w) or agar (1% w/w) in an aqueous sodium benzoate solution at the desired concentration. The mixture was heated with continuous stirring until homogenized. For gelatin, the temperature was maintained below 45 °C to prevent denaturation. The resulting solution (18 mL) was poured into a custom-built 2D plexiglass reactor (Figure 1), designed with a thin circular gel layer (0.7 mm thick, 8.8 cm outer diameter, 1.8 cm inner diameter). After sealing, the gel was set for gelation (2 h for agar, 24 h for gelatin). To initiate crystallization, the gel within the inner circle was removed to create a reaction-diffusion interface, and 3 mL of outer HCl solution (1.00 M) was added.

**Figure 1:**
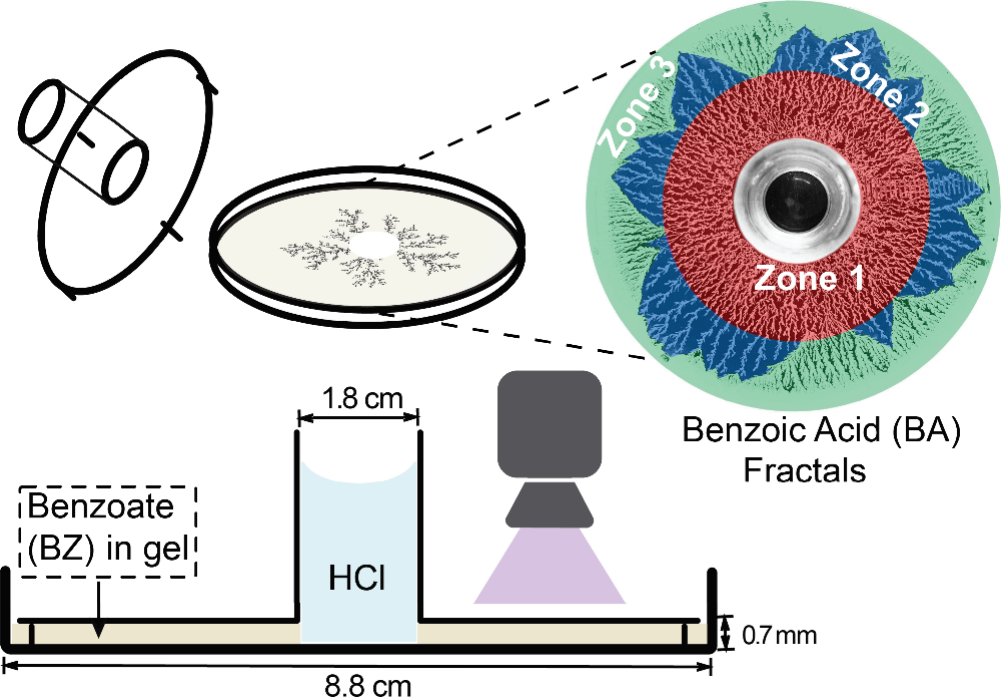
Schematic representation of the experimental setup used to study BA crystallization in RDF. The system consists of a gelatin or agar gel matrix containing an inner benzoate (BZ) solution, with an outer H^+^ source initiating diffusion-driven crystallization. The schematic illustrates the reactor geometry, diffusion interface, and controlled conditions that enable systematic investigation of fractal growth, gel-matrix interactions, and crystallization pathways. The panel is a color-coded representation of **Zones 1–3**. The red, blue, and green colors correspond to **Zones 1, 2, and 3**, respectively.

Temperature control was critical for our system as temperature influences the crystallization outcomes by affecting solubility, nucleation kinetics, and transport properties. For example, increasing the temperature increases the solubility of benzoic acids, shifting the supersaturation threshold and delaying nucleation.^20^ Simultaneously, it increases both the rate of diffusion of ions and the reaction while altering the viscoelastic property of the gel media.^21–23^ To ensure reproducibility and fair comparison between experiments with different inner ion concentrations, the temperature of the gel was maintained at 19 ± 1 °C throughout the gelation period and reaction.

### Characterization

Fractal growth was monitored via optical imaging using a Canon EOS 700D camera equipped with a macro lens (EF-S 60 mm) at fixed time intervals. The crystals were carefully washed with warm deionized water, air-dried, and coated with a 5 nm gold film for scanning electron microscopy (SEM) analysis. The purified crystals were further washed in warm water, isolated via centrifugation, and freeze-dried for powder X-ray diffraction (PXRD) analysis. XRD patterns were recorded on a Bruker D8 Advance Diffractometer using Cu–Kα radiation (40 kV, 40 mA, step size 0.02°).

### Fractal Dimension

The fractal dimension (D) of benzoic acid (BA) fractals was determined using the box-counting method via the FracLac plugin in ImageJ. Fractal images were first thresholded to binary, and multiple grid sizes were overlaid to compute ln *N*(ε) vs ln (ε), where *N*(ε) represents the number of occupied boxes of size ε. The negative slope of this plot yields the fractal dimension (Figure S1).

To ensure accuracy, the method was validated using well-established fractal structures with known dimensions, including the Koch snowflake (*D* = 1.2618) and Sierpinski triangle (*D* = 1.585),^24^ producing measured values of *D* = 1.277 ± 0.024 and *D* = 1.575 ± 0.035, respectively, confirming the reliability of our approach.

For BA fractals, the fractal dimension is not uniform across the entire structure due to morphological variations arising from the reaction-diffusion process. Additionally, the concentric ring geometry of the reactor presents challenges in directly computing the fractal dimension across the full pattern. To address this, multiple rectangular subregions aligned tangentially to the radial fractal were analyzed separately and then averaged. Rectangular sampling windows were chosen to approximate channel geometry, allowing box-counting to be performed along locally linear segments. The fractal dimension was computed across a range spanning approximately 1.5–3 orders of magnitude in box size. Given the self-similar nature of fractals, computing the fractal dimension radially or in rectangular sections yields equivalent results, similar to DLA clusters grown in radial versus channel or square geometries.^1,25,26^

To ensure statistical rigor, the final fractal dimension was determined using an inverse-variance-weighted average, which minimizes variance and maximizes the likelihood of approximating the accurate fractal dimension with optimized weighting. Additionally, correlation coefficients (*r* ^2^) were computed for each fit to reduce the effect of outliers, and measurements with *r* ^2^ < 0.98 were excluded from the final analysis.

## Results and Discussion

Fractal growth of BA crystals in reaction-diffusion conditions revealed two distinct morphological regimes, depending on the gel network. In gelatin-based systems, BA formed dendritic fractals closely resembling diffusion-limited aggregation (DLA) clusters, whereas in agar-based systems, BA crystallized into spherulitic structures. The difference in growth patterns suggests that gel network chemistry plays a fundamental role in modulating crystal growth under diffusion constraints.

### Fractal Morphologies in Gelatin

In gelatin-based systems, BA crystallization followed a dendritic growth pattern resembling diffusion-limited aggregation (DLA) (Figure 2). As H^+^ ions diffused into the gelatin gel containing benzoate, fractal growth patterns emerged, progressing outward in a manner governed by diffusion constraints. The observed fractal structures were classified into three distinct growth zones based on their morphology: **Zone 1**, located in the inner part close to the interface, exhibited a fine dendritic pattern, characteristic of diffusion-limited growth. As crystallization progressed outward, a critical transition point was observed at higher benzoate concentrations, giving rise to **Zone 2**, where the fractal branches thickened and widened, and the internodal distance (distance between successive branch points) increased. This transition was particularly evident at moderate to high inner [BZ] concentrations (≥0.10 M), where increased supersaturation led to a denser aggregation pattern. Beyond this region, a dense bifurcation forms **Zone 3**, characterized by extensive crystal deposition predominantly on the reactor surface, leading to a densely packed structure. In this zone, the fractal nature is significantly diminished or becomes immeasurable due to reduced void space.

**Figure 2:**
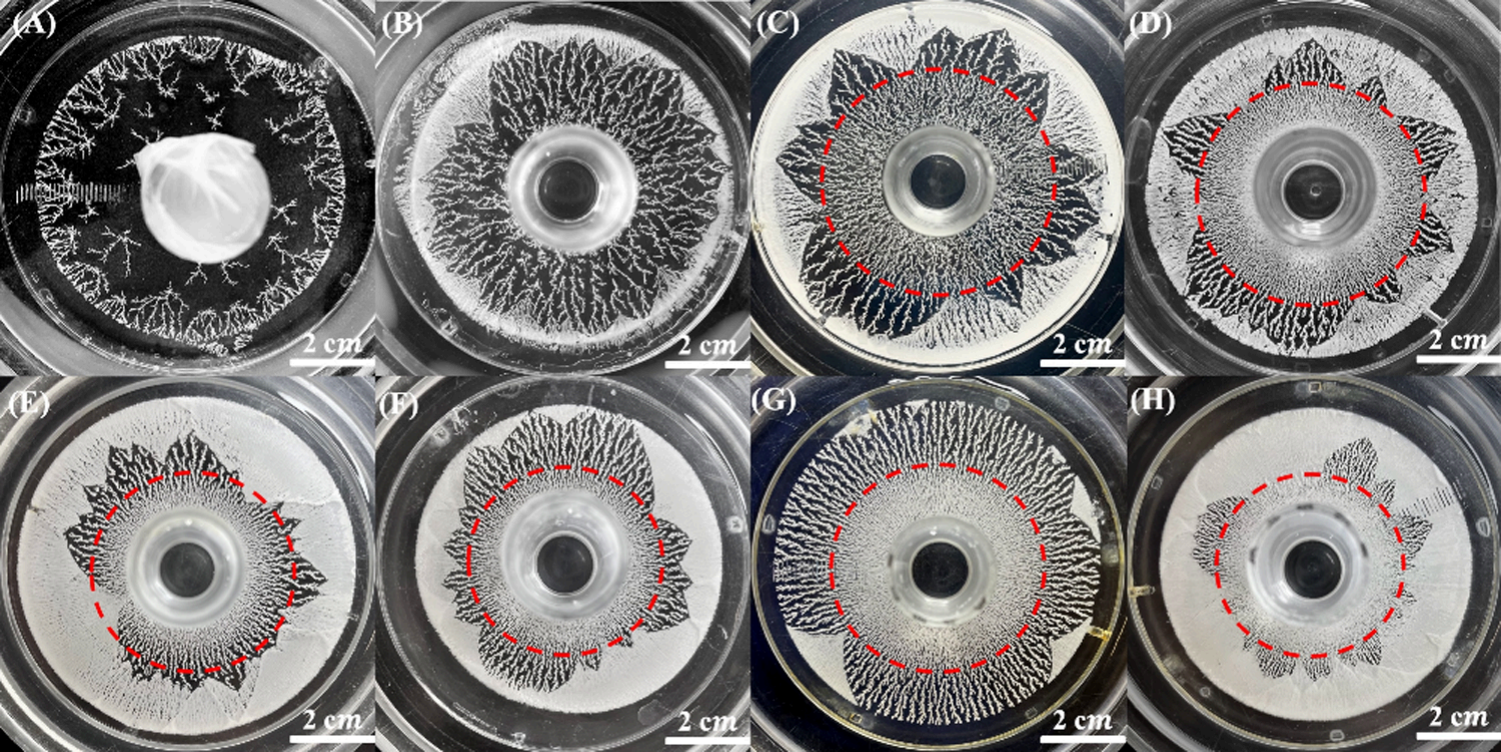
Optical images of benzoic acid fractals formed in the gelatin-based reaction-diffusion system after 3 days of diffusion, with a constant outer [H^+^] = 1.0 M and varying inner [BZ]: (A) 0.05 M, (B) 0.08 M, (C) 0.10 M, (D) 0.12 M, (E) 0.14 M, (F) 0.16 M, (G) 0.18 M, and (H) 0.20 M. The observed transition from highly branched dendritic structures to thicker, more compact aggregates with increasing [BZ] reflects the influence of supersaturation on fractal morphology. The red contour marks the boundary between **Zone 1** and **Zone 2**, highlighting the transition from densely branched dendrites to structures with reduced connectivity.

The effect of inner [BZ] concentration on these morphological transitions is illustrated in Figure 2, which shows BA fractals formed in gelatin-based systems with the outer [H^+^] concentration fixed at 1.0 M. At lower inner [BZ] concentrations (0.05 and 0.08 M), the reduced supersaturation resulted in a lower nucleation density, leading to sparse crystallite formation. This inhibited the development of well-defined fractal transitions, yielding a more open and less interconnected structure. In contrast, at higher inner [BZ] concentrations (≥0.10 M), increased nucleation events promoted clear morphological transitions, reinforcing the role of supersaturation-driven diffusion-limited aggregation in governing fractal growth dynamics.

SEM analysis of the three growth zones further elucidates the influence of gelatin network confinement on benzoic acid crystallization (Figure 3). Despite the macroscopic variations observed between **Zones 1, 2, and 3**, the microscopic morphology of individual crystals remained consistent across all regions, with needle-like structures dominating the crystallization pattern. The measured needle widths were 478 ± 125 nm in **Zone 1**, 441 ± 79 nm in **Zone 2**, and 544 ± 70 nm in **Zone 3**. These values correlate well with the pore size of the gelatin network (320–650 nm),^27^ reinforcing the role of gelatin’s confinement effect in controlling crystal growth.

**Figure 3:**
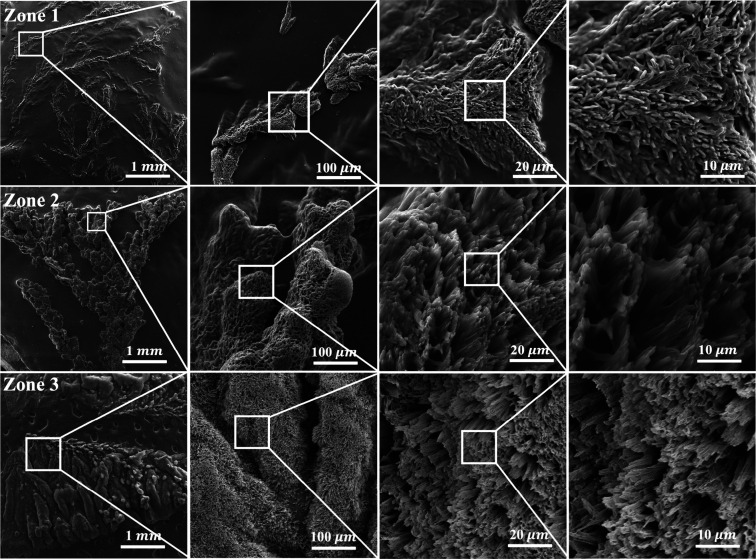
SEM images at different magnifications of BA crystals in **Zone 1–3** of the gelatin-based reaction-diffusion system formed at an inner [BZ] of 0.10 M. In **Zone 1**, the images reveal densely branched dendritic structures characteristic of diffusion-limited aggregation (DLA), with fine, well-defined crystal branches indicative of rapid nucleation and diffusion-controlled growth. In **Zone 2**, the crystal structures exhibit thicker branches with reduced secondary branching compared to **Zone 1**, indicating a transition from highly branched diffusion-limited growth to more compact aggregation as supersaturation and nucleation rates decrease. Unlike **Zone 1** and **Zone 2**, **Zone 3** exhibits densely packed, surface-attached crystallization with minimal fractal characteristics. The compact morphology suggests a transition from diffusion-controlled growth to surface-mediated deposition, with increased mechanical constraints leading to localized strain accumulation.

The observed increase in crystal dimensions in **Zone 3** is likely due to a transition toward surface-mediated deposition. Due to the dense attachment of crystals in all zones, the precise measurement of individual crystal sizes was not feasible, as overlapping structures prevented the isolation of single complete crystals. These findings highlight that while the reaction-diffusion environment dictates the fractal architecture on a macroscopic scale, the gelatin matrix exerts a uniform influence on microscopic crystal morphology.

Powder X-ray diffraction (PXRD) analysis was conducted to assess the phase purity and structural characteristics of benzoic acid crystals across the three growth zones (Figure 4). The diffraction patterns for **Zones 1, 2, and 3** closely matched the simulated PXRD pattern, confirming that all regions share the same crystalline phase, with morphological differences arising purely from growth dynamics rather than polymorphism or chemical impurities. The strongest diffraction peaks at 8.1–8.2° (002), 17.2–17.3° (10 $\overset{-}{1}$ ), and 23.9–24.1° (014) were present across all zones. Also, the crystallite sizes calculated by applying the Scherrer equation onto the full width at half-maximum (Table S1) from the XRD peaks^28,29^ for the major reflections—(002), (10 $\overset{-}{1}$ ), and (014)—showed no significant change across the zones: **Zone 1** (36, 37, and 36 nm), **Zone 2** (39, 41, and 41 nm), and **Zone 3** (36, 36, and 36 nm).

**Figure 4:**
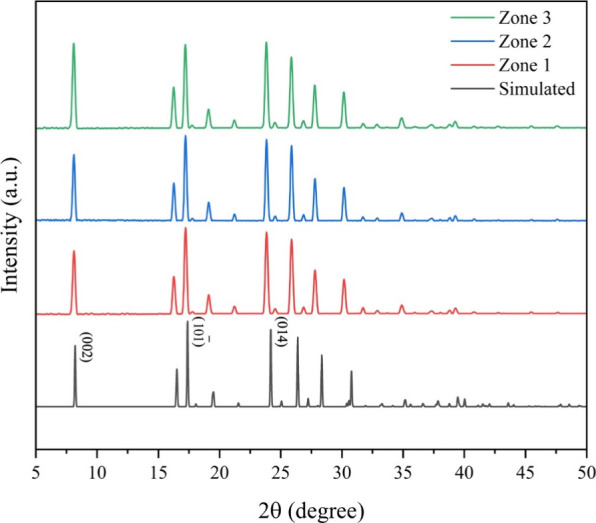
PXRD patterns of BA crystals from **Zones 1, 2, and 3** in the gelatin-based reaction-diffusion system, compared to the simulated PXRD pattern of BA. The diffraction peaks confirm phase purity across all zones, and the peak shifts at high 2θ indicates the presence of macrostrain.

When the peaks of the three zones were compared to those of the simulated pattern, a left shift that became more prominent at higher 2θ was observed across all three zones (Figure S2). This shift indicates the presence of uniform tensile macrostrain across all growth zones,^30,31^ which is consistent with the fractal crystallization dynamics of benzoic acid under diffusion-limited conditions. The formation of dendritic and branched fractals through DLA and in the context of RDF involves rapid, nonequilibrium growth at the diffusion front. Molecules attach preferentially at the outer tips of the structure, where they have limited time to rearrange into their lowest-energy lattice configurations. This kinetically driven attachment process can embed local lattice distortions, which accumulate and manifest as macrostrain across the growing structure.^32,33^ In addition, the intrinsic multiscale, open nature of fractal assemblies creates spatially heterogeneous packing, where neighboring domains may exhibit slight misalignment or inconsistent packing density. Such irregularities in internal connectivity can produce anisotropic stress fields that become frozen into the lattice during growth.^34,35^ On the other hand, the gel matrix imposes mechanical resistance to crystallization, especially in later stages as the crystals expand and encounter confinement. In both gelatin and agar systems, the gel network restricts structural relaxation and defect mobility, potentially inducing tensile stress as the growing crystals attempt to expand against the matrix.^36^ Taken together, these experimental and theoretical insights support the conclusion that the observed tensile macrostrain is not a zone-specific anomaly but a systemic outcome of the fractal morphogenesis process under reaction-diffusion control. The persistent shift across all zones likely reflects lattice distortions embedded during fast, constrained growth, further amplified by the mechanical resistance of the gel matrix and, in later stages, by surface attachment effects at the reactor boundary.

### Fractal Dimension in Gelatin

The fractal dimension analysis provides further insight into the growth dynamics of benzoic acid (BA) fractals in gelatin, aligning well with the macroscopic, microscopic (SEM), and structural (PXRD) observations. Images of the fractals produced at varying inner [BZ] (0.05–0.22 M) at constant outer [H^+^] (1.0 M) were analyzed to quantify their self-similarity and aggregation behavior (Figure 5 and Table 1). The results indicate a clear dependence of fractal dimension on inner [BZ], with a gradual increase in fractal dimension as [BZ] increases. At higher inner [BZ], however, the variation in fractal dimension became minimal, converging toward a theoretical limit.

**Table 1:** Fractal Dimensions (*D*) and Their Respective Uncertainties for **Zone 1** and **Zone 2** of BA Fractals in the Gelatin-Based Reaction-Diffusion System^a^

inner [BZ] (M)	fractal dimension of Zone 1	fractal dimension of Zone 2
0.05	1.56 ± 0.02	-^b^
0.08	1.66 ± 0.03	-^b^
0.10	1.71 ± 0.02	1.55 ± 0.03
0.12	1.73 ± 0.02	1.63 ± 0.02
0.14	1.72 ± 0.02	1.66 ± 0.02
0.16	1.73 ± 0.04	1.69 ± 0.01
0.18	1.74 ± 0.04	1.69 ± 0.02
0.20	1.74 ± 0.05	1.71 ± 0.05

a The data highlights the trend of increasing *D* with inner [BZ], with **Zone 1** exhibiting higher fractal dimensions characteristic of DLA, while **Zone 2** displays lower values due to reduced branching and increased aggregation density.

b **Zone 2** did not appear.

**Figure 5:**
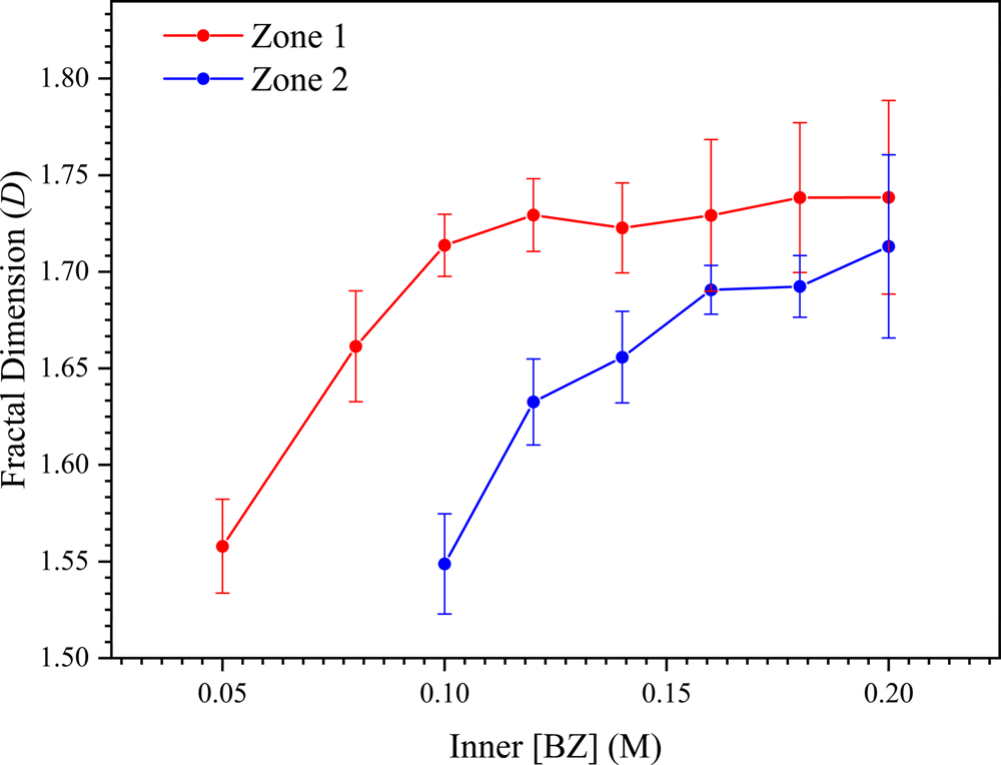
Graph of fractal dimension (*D*) versus inner [BZ] (M) for **Zone 1** and **Zone 2** in the gelatin-based reaction-diffusion system. The data show an increase in fractal dimension with rising inner [BZ], with values converging toward the classical DLA limit in **Zone 1**. In **Zone 2**, fractal dimensions remain consistently lower, reflecting the transition from highly branched dendritic structures to thicker, less interconnected aggregates as supersaturation and diffusion rates decrease.

For **Zone 1**, the lowest recorded fractal dimension was 1.56 ± 0.03 at [BZ] = 0.05 M, with values increasing as inner [BZ] increased, eventually converging to ∼ 1.74, which is close to the classical diffusion-limited aggregation (DLA) limit of 1.71 ^5,7,25,37–39^ for uniform particle size.^40^

**Zone 2** followed a similar trend, with fractal dimensions increasing progressively with inner [BZ] starting at 1.55 ± 0.02 but displaying consistently lower values than **Zone 1**. This right-shifted behavior indicates a gradual transition from densely branched dendritic structures (**Zone 1**) to sparser patterns with thicker branches (**Zone 2**), consistent with the macroscopic morphological changes shown in Figure 2. In contrast, **Zone 3** exhibited a densely packed structure that prevented reliable measurement of the fractal dimension, as void spaces were nearly entirely occupied by aggregated crystallites. This dense aggregation supports the interpretation from SEM and PXRD analyses, suggesting a transition from diffusion-controlled fractal growth to compact, surface-mediated crystal deposition.

These fractal dimension trends align with SEM findings, where **Zone 1** displayed the finest, most branched dendritic structures, while **Zone 2** exhibited thicker branches with reduced secondary branching. The convergence of fractal dimension toward 1.71–1.74 at higher inner [BZ] suggests that fractal growth approaches an idealized DLA regime, where aggregation is dominated by diffusion constraints rather than kinetic effects.

The reaction-diffusion framework (RDF) governing BA precipitation plays a key role in determining fractal evolution. Given that BA precipitation is driven by a rapid neutralization reaction,^41^ the reaction rate is significantly faster than diffusion, making this system a clear example of diffusion-limited precipitation (DLP).^42^ This was confirmed experimentally by adding bromocresol green as a pH indicator, where the precipitation front closely followed the acid diffusion front, demonstrating that diffusion constraints primarily govern the fractal morphogenesis in this system (Supporting Information).

Thus, the integration of fractal dimension analysis with SEM and PXRD findings highlights a consistent growth mechanism where **Zone 1** and **Zone 2** follow a DLA-like self-similar aggregation model, while **Zone 3** transitions into a dense, surface-constrained crystallization mode with increased strain effects and preferred orientation. This study reinforces the universality of DLA principles in molecular crystallization, further demonstrating how reaction-diffusion conditions can be leveraged to control fractal growth morphology in soft-matter systems.

### Computer Simulations

To interpret and quantitatively support the experimental findings of BA fractal growth in gelatin, we developed a Monte Carlo-based diffusion-limited aggregation (DLA) simulation in MATLAB that mimics the RDF crystallization system. Our simulation adopts the standard DLA simulation, where random-walking particles were initialized at the outer boundary and diffused toward the inner circle, attaching to a growing aggregate upon contact, with the extension such as grid designed in a concentric circular geometry, closely resembling the 2D experimental reactor. The aggregation process was governed by a tunable sticking coefficient (*S*), defined as the probability of a moving particle irreversibly adhering to the growing cluster at the center, which controlled the branching density and overall fractal morphology. The fractal dimension of the simulated aggregates was computed using the box-counting method, allowing direct comparison with experimentally measured fractal dimensions from benzoic acid crystallization in gelatin.

The Monte Carlo simulation results closely paralleled experimental trends, confirming that stochastic growth mechanisms dominate fractal formation in the benzoic acid reaction-diffusion system. Consistent with previous DLA studies,^43,44^ our simulation revealed an inverse relationship between the fractal dimension (*D*) and the sticking coefficient (*S*): lower sticking coefficients produced aggregates with higher fractal dimensions (Table 2). When the fractal dimension of the simulated aggregates was plotted against −log (*S*), the resulting curve (Figure 6) showed a striking resemblance to the experimental fractal dimension vs inner [BZ] graph (Figure 5). This correlation suggests that inner [BZ] in the experimental system effectively modulates the sticking coefficient in the crystallization process, indicating that supersaturation-driven nucleation affects local attachment probabilities similarly to a Monte Carlo-controlled DLA model.

**Table 2:** Sticking Coefficients (*S*) and Their Corresponding Fractal Dimensions (*D*) Obtained from Monte Carlo Simulations of DLA^a^

sticking coefficient, *S*	fractal dimension, *D*
0.05	1.701 ± 0.002
0.075	1.697 ± 0.003
0.1	1.691 ± 0.009
0.125	1.691 ± 0.003
0.15	1.686 ± 0.011
0.175	1.681 ± 0.013
0.2	1.671 ± 0.011
0.225	1.667 ± 0.007
0.25	1.662 ± 0.008
0.275	1.651 ± 0.002
0.3	1.636 ± 0.010
0.325	1.612 ± 0.011
0.35	1.571 ± 0.003

a The data illustrates the inverse relationship between *S* and *D*, where lower sticking coefficients lead to more highly branched fractal structures with higher fractal dimensions, while higher *S* values result in thicker, less branched aggregates.

**Figure 6:**
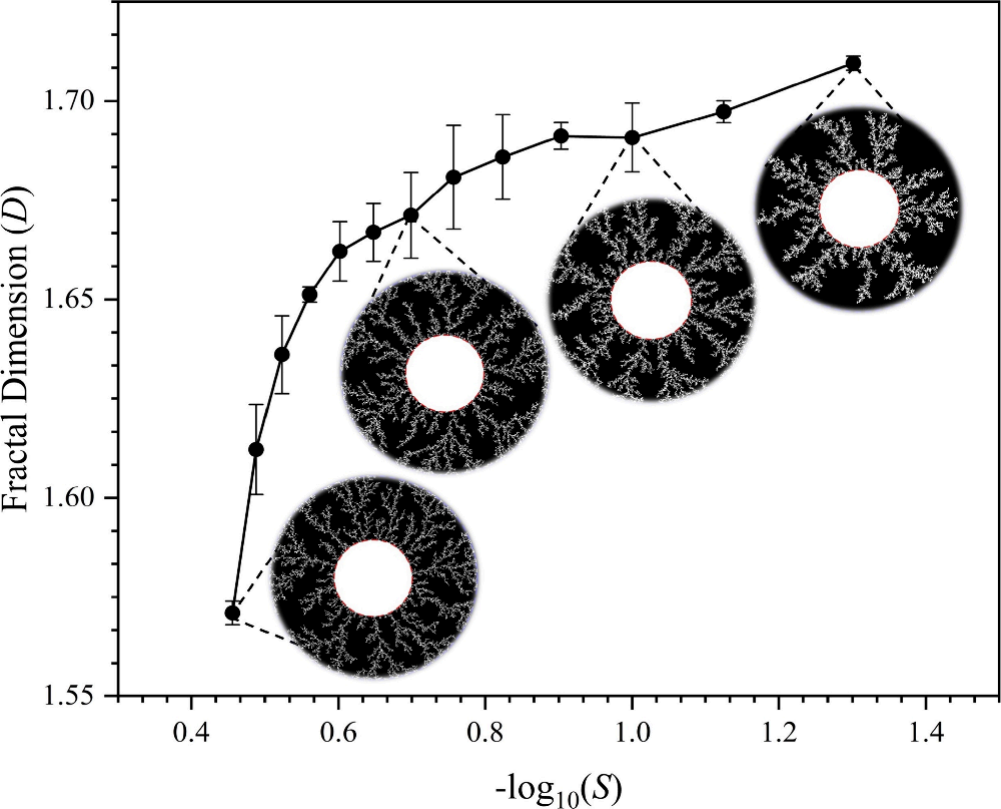
Graph of fractal dimension (*D*) versus −log_10_(*S*) for simulated diffusion-limited aggregation (DLA) fractals, illustrating the inverse relationship between the sticking coefficient (*S*) and fractal complexity. Insets show representative DLA fractal structures generated from numerical simulations at different *S* values (*S* = 0.35, 0.20, 0.10, and 0.05 from left to right), demonstrating the transition from sparse, thick-branching aggregates at high *S* to highly branched, dendritic structures at low *S*.

For example, lower sticking coefficients (corresponding to higher inner [BZ]) lead to higher fractal dimensions corresponding to denser dendritic structures with more frequent secondary branching. This behavior aligns with SEM observations, where **Zone 1** exhibited the most secondary branching, a feature replicated in low-*S* Monte Carlo DLA simulations. Conversely, at high sticking coefficients (or low inner [BZ]), the growth of branches becomes sparer, a trend consistent with the reduced fractal dimension in **Zone 2**, where SEM images show branches with lower connectivity that grow in an opening manner. These findings confirm that the experimental system exhibits a diffusion-limited to surface-dominated growth transition, analogous to the behavior captured in Monte Carlo-based stochastic aggregation models. By demonstrating that inner [BZ] modulates effective sticking probability in crystal aggregation, this study bridges the gap between reaction-diffusion crystallization and classical DLA models, reinforcing the universality of stochastic self-assembly principles in molecular crystallization.

To further explore the experimental significance of sticking coefficient variations, we examined the relationship between diffusion, supersaturation, and fractal transitions in benzoic acid crystallization within gelatin. Looking at Figure 5, the observed decrease in fractal dimension from **Zone 1** to **Zone 2** can be attributed to changes in diffusion and supersaturation rate. In a reaction-diffusion framework (RDF) within a gel medium, the size of precipitated crystals generally increases along the diffusion direction because the concentration gradient of the outer reactant (H^+^) flattens over time, reducing the extent of supersaturation.^10,12,45,46^ With this decrease in supersaturation, nucleation slows down, and crystal growth becomes dominant, establishing an increasing crystal size gradient along the diffusion front.^11^ Several studies have demonstrated that fractal dimension decreases with increasing particle size in DLA models,^37,38,40^ supporting the idea that the larger crystallites observed in **Zone 2** contribute to the reduction in fractal dimension. The increase in crystal size enhances the likelihood that neighboring crystals adhere to one another, effectively behaving as an increased sticking coefficient scenario, thus contributing to thicker branches and reduced fractal dimension observed in **Zone 2**. This phenomenon also explains the observed decrease in the transition radius (the radial distance from the initial diffusion interface to the onset of **Zone 2**) as inner [BZ] increases (Figure 7). As the reaction progresses outward, the supersaturation gradient becomes flatter and reduces local nucleation rates, favoring the growth and aggregation of existing crystals. Such diffusion-controlled growth conditions and the associated morphological transitions were further corroborated by Monte Carlo simulations, which demonstrated enhanced branching density at higher diffusivities and lower sticking coefficients,^47^ aligning closely with the experimentally observed differences between **Zone 1** and **Zone 2**.

**Figure 7:**
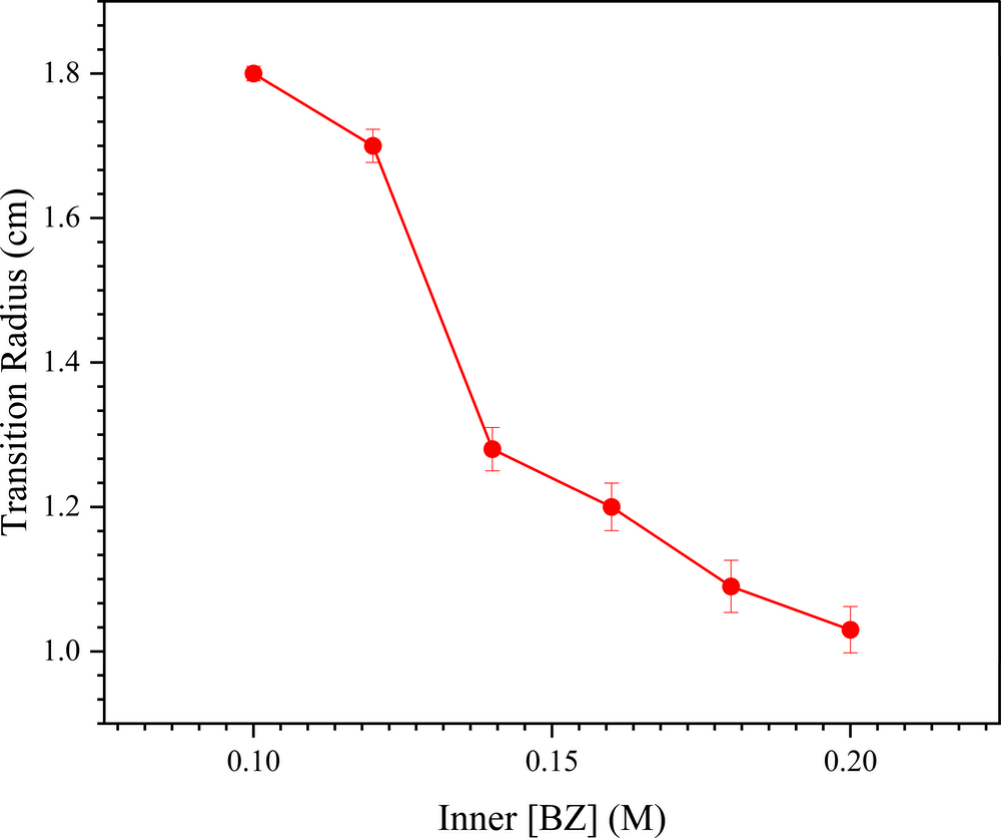
Dependence of the transition radius (cm), defined as the radial distance from the diffusion interface to the onset of **Zone 2**, on the inner [BZ] (M) in the gelatin-based reaction-diffusion system. The observed decrease in transition radius with increasing inner [BZ] reflects changes in local supersaturation gradients and diffusion rates, highlighting their role in modulating fractal growth transitions.

However, while diffusional changes explain a gradual decrease in fractal dimension, they do not fully account for the sudden transition between **Zone 1** and **Zone 2** observed in Figure 2. Instead, we propose that this abrupt change is linked to pH-dependent modifications in the gelatin network. As the [H^+^] concentration wave propagates outward, the pH of the medium increases, affecting the ionization state of the gelatin’s amino acids. Gelatin, derived from collagen, contains many ionizable functional groups, which undergo protonation or deprotonation depending on the surrounding pH. Goudie et al. demonstrated that the helical content of gelatin decreases sharply below pH 5, which falls within the pH range of our system.^48^ Since gelatin plays a central role in structuring the reaction-diffusion environment, a critical pH threshold could alter its electrochemical interactions with benzoate ions, affecting both crystal growth dynamics and fractal morphology. At this critical pH, gelatin may restructure or cross-link differently, leading to a sudden shift in the sticking coefficient, manifesting as the transition from **Zone 1** to **Zone 2**.

To further validate this relationship between supersaturation and fractal transitions, a MATLAB simulation was conducted using the same Monte Carlo DLA model but with a sudden increase in sticking coefficient at a specific radial distance (Figure 8). The resulting fractal structures exhibited a clear transition between two distinct fractal regimes, resembling the transition from **Zone 1** to **Zone 2** in the benzoic acid fractals. The fan-like macro branches of the outer zone (lower *S*) resemble the widening of branches observed at Zone 2. When the fractal dimensions were compared, a similar sudden drop was observed, supporting the argument that changes in supersaturation and diffusion drive effective sticking coefficient variations in the experimental system. This comparison confirms a strong connection between diffusion-limited aggregation and reaction-diffusion precipitation, where modulations in supersaturation and pH-dependent gel interactions dictate crystal aggregation patterns.

**Figure 8:**
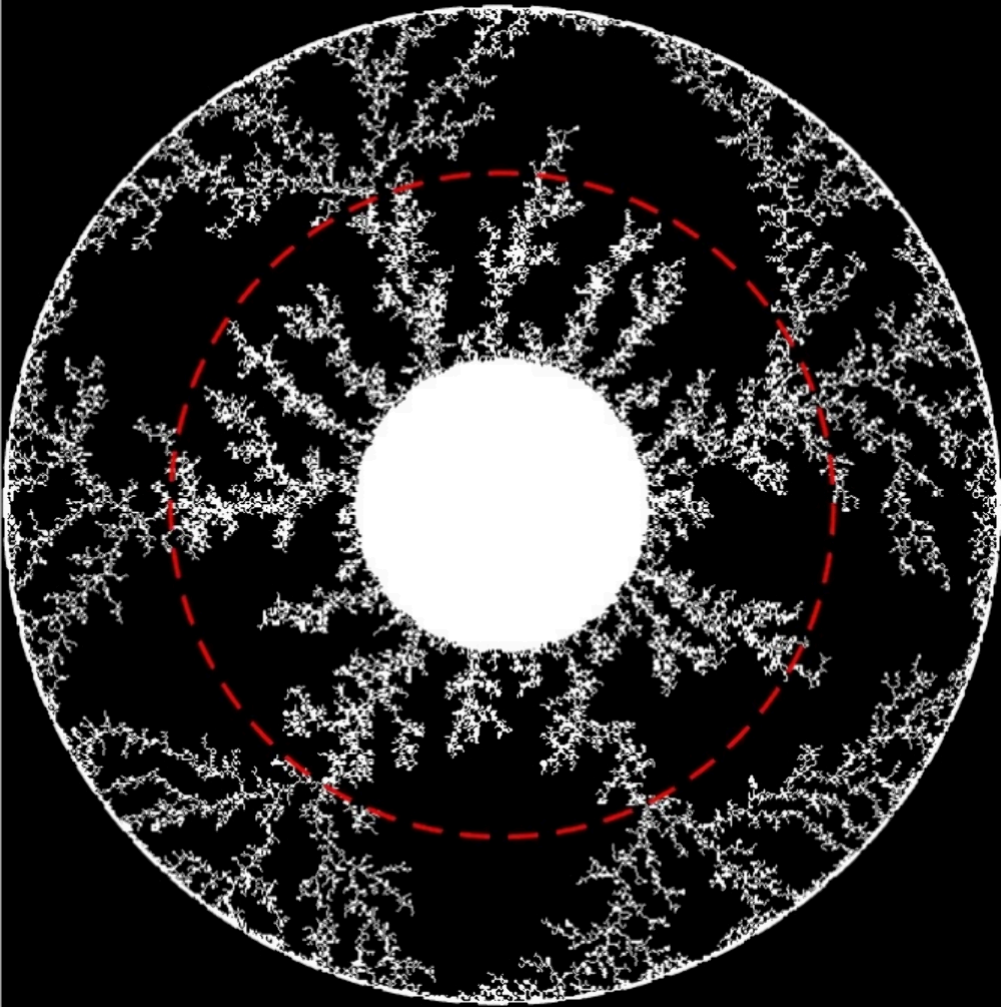
Monte Carlo simulation snapshot depicting BA fractal structures generated via diffusion-limited aggregation (DLA) with an abrupt increase in the sticking coefficient (*S*) from 0.05 to 0.25 at a specific radial distance (marked by the red dashed circle). This sudden change illustrates a morphological transition from densely branched dendrites (low *S*) to thicker, sparsely branched aggregates (high *S*), closely mirroring experimental observations and emphasizing the critical role of local variations in attachment probability on fractal morphogenesis.

The formation of **Zone 3**, characterized by dense crystallization on the reactor surface, remains a complex phenomenon influenced by multiple interrelated factors. One possible explanation is that **Zone 2** facilitates the emergence of **Zone 3** by promoting surface attachment. In most cases, **Zone 2** and **Zone 3** appear simultaneously, suggesting a structural link between them. As observed in SEM images, the thickening of branches in **Zone 2** could enhance surface attachment, particularly in the confined 0.7 mm gel layer. The mean branch thickness in **Zone 1** (∼0.35 mm) increases to ∼0.5 mm in **Zone 2**, supporting the idea that bulkier structures promote contact with the reactor surface, thereby initiating compact crystallization in **Zone 3**. An alternative explanation is that **Zone 3** emerges due to the intrinsic behavior of gelatin-based reaction-diffusion systems. Previous studies on reaction-diffusion precipitation in gelatin, such as the work of Dayeh et al.,^49^ demonstrated the formation of star-like bifurcation patterns resembling the morphology of **Zone 3** in our system. When the experiment was repeated without the outer reactor, a similar bifurcation zone appeared at the gel–air interface (Figure S3), indicating that **Zone 3**-like transitions could be inherent to gelatin-mediated reaction-diffusion crystallization. Taken together, these arguments suggest that **Zone 3** arises from a combination of factors: the physical influence of **Zone 2**, inherent reaction-diffusion dynamics in gelatin, and surface attachment constraints that alter crystallization behavior at the interface.

### Effect of Diffusion Coefficient on Fractal Growth

A reverse-phase system was examined to investigate further the role of diffusion dynamics in fractal formation, where HCl was placed in the inner gel phase, and sodium benzoate was allowed to diffuse inward from the outer solution. This setup reverses the direction of reactant diffusion compared to the normal-phase system, allowing us to assess how variations in diffusion coefficients affect fractal morphology. According to the Stokes–Einstein equation,^50^ the diffusion coefficient of an ion is inversely related to its ionic radius. Since benzoate ions (C_6_H_5_COO^–^) have a significantly larger ionic radius than H^+^ ions, their diffusion in gelatin is much slower, providing a controlled way to probe the effect of reduced diffusion rates on fractal growth.

Figures S4 and S5 show the benzoic acid fractals formed in 2D and 1D reactors under this reverse-phase condition. Compared to the normal-phase system, the fractal structures in the 2D reactor exhibited apparent thickening of the branches and a reduction in fractal dimension from 1.71 ± 0.02 (normal-phase) to 1.66 ± 0.04 (reverse-phase). Also, crystal growth on the reactor surface in the 2D system occurred closer to the inner circle, indicating that slower diffusion constraints alter fractal morphogenesis.

These results are analogous to the transition from **Zone 1** to **Zone 2** in the normal-phase system, strongly supporting the idea that the slowdown of the diffusion rate is a key factor in the shift toward thicker branches and reduced fractal dimension. In both cases, as diffusion rates decrease, growth becomes more compact, and branching density is reduced. The reverse-phase system, therefore, reinforces the interpretation that the transition from **Zone 1** to **Zone 2** in the normal-phase system arises due to decreasing supersaturation and diffusion rate, highlighting the critical role of diffusion-limited aggregation in dictating fractal evolution in reaction-diffusion crystallization.

### Fractal Morphologies in Agar

In contrast to the gelatin-based reaction-diffusion system, where BA fractals exhibit dendritic growth resembling DLA, using agar as the gel network resulted in an entirely different fractal morphology. Instead of the highly branched structures characteristic of DLA, BA crystallization in agar led to spherulitic crystal growth, as shown in Figure 9. PXRD analysis confirmed that crystals formed in agar (Figure S6) share the same BA crystalline phase as those in gelatin, highlighting the significant role of gel-network chemistry in crystallization pathways. The crystallite sizes calculated using the Scherrer equation for the primary reflections (002), (10 $\overset{-}{1}$ ), and (014), were 54.4, 53.9, and 45.6 nm, respectively. These crystallite sizes are notably larger compared to the gelatin system, indicating reduced lattice strain under agar-mediated crystallization conditions. Moreover, the relatively smaller crystallite size observed for the (014) reflection suggests a preferred crystallographic orientation induced by the agar matrix, highlighting the gel’s influence on crystal growth anisotropy.^51^ However, despite the distinct morphologies, both agar and gelatin systems exhibited sudden transitions in fractal structure, delineating three different zones at a critical distance from the inner circle and marking changes in growth dynamics as diffusion progresses.

**Figure 9:**
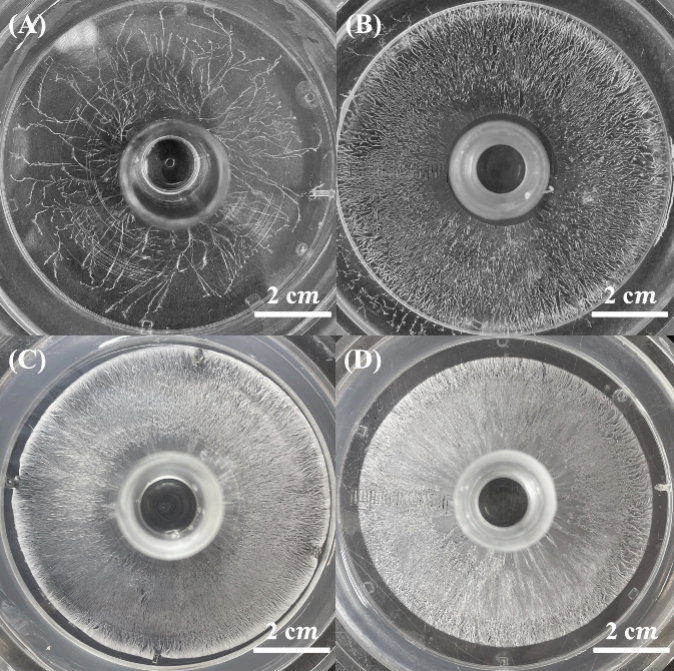
Optical images of BA fractal structures formed in the agar-based reaction-diffusion system after 3 days of diffusion, with constant outer [H^+^] = 1.0 M and varying inner [BZ]: (A) 0.05 M, (B) 0.10 M, (C) 0.15 M, and (D) 0.20 M. Increasing inner [BZ] results in noticeable morphological transitions, progressing from sparse spherulitic clusters at lower concentrations to densely interconnected fractal aggregates at higher concentrations, highlighting the significant role of initial supersaturation in governing fractal pattern formation.

To further analyze these morphological differences, the three distinct regions (**Zones 1, 2, and 3**) were examined using optical microscopy (Figure 10) and SEM (Figure 11), revealing clear variations in crystal aspect ratios and sizes. The average projected surface area of crystals, measured from the SEM image (Figure 11), increased progressively from **Zone 1** to **Zone 3** (Table 3), consistent with expectations based on decreasing supersaturation along the diffusion front.^10,45,46^ However, unlike gelatin, agar exhibited a distinctly different evolution in crystal morphology. In **Zone 1**, BA crystals appeared as rectangular plates with an aspect ratio of approximately 0.60, forming web-like stacked networks. In contrast, **Zone 2** and **Zone 3** exhibited elongated dendritic and irregular thread-like aggregates, respectively, indicating a fundamentally different morphological evolution compared to the gelatin system.

**Table 3:** Average Aspect Ratios and Projected Surface Areas of Benzoic Acid Crystals Measured from SEM Images in **Zones 1–3** of the Agar-Based Reaction-Diffusion Aystem at Inner [BZ] = 0.20 M^a^

Zone #	average aspect ratio	average surface area (μm^2^)
**Zone 1**	0.603	796
**Zone 2**	0.088	2167
**Zone 3**	0.320	11,180

a Differences in crystal shape and size metrics across zones reflect changing growth conditions driven by variations in local supersaturation, diffusion constraints, and gel-matrix interactions.

**Figure 10:**
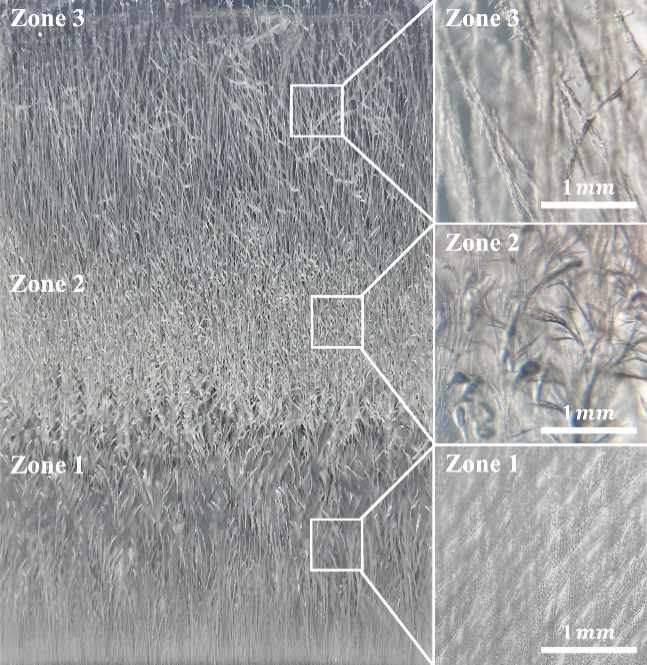
Optical microscopy images (40× magnification) illustrating the morphological evolution of BA crystals in **Zones 1–3** within an agar-based reaction-diffusion system at an inner [BZ] of 0.15 M. **Zone 1** shows stacked rectangular plate-like structures, **Zone 2** exhibits elongated dendritic features with characteristic branching, and **Zone 3** presents irregular, thread-like aggregates, reflecting distinct crystallization dynamics driven by decreasing supersaturation and diffusion constraints along the diffusion fronts.

**Figure 11:**
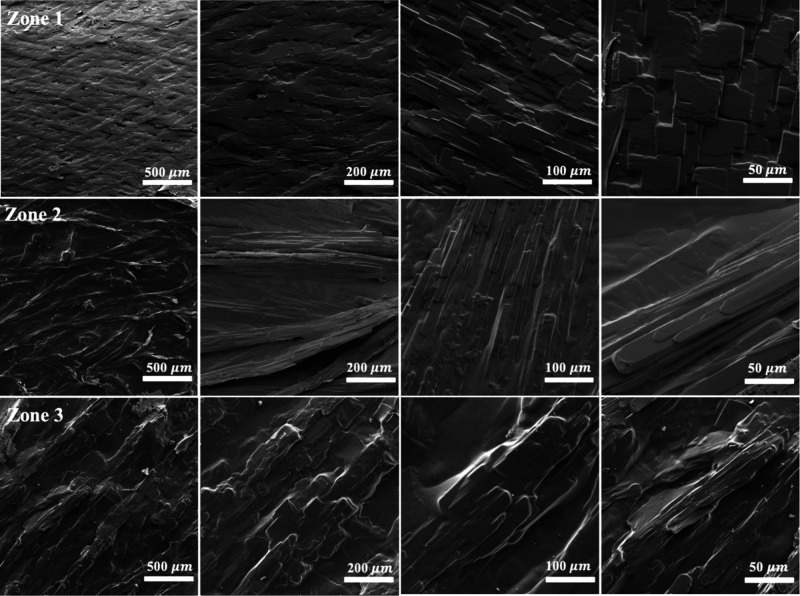
SEM images of BA crystals formed in the agar-based reaction-diffusion system at an inner [BZ] concentration of 0.15 M. Images illustrate morphological variations across the three distinct growth zones at different scales. **Zone 1** shows densely stacked rectangular plate-like crystals, **Zone 2** reveals elongated dendritic structures indicative of tip-splitting growth, and **Zone 3** exhibits irregular thread-like aggregates with less defined, more compact morphologies. These morphological transitions highlight the influence of changing local supersaturation, diffusion dynamics, and gel-matrix interactions as crystallization progresses outward from the reaction interface.

Despite the stark morphological differences between gelatin and agar systems, both gels share a key functional role in reaction-diffusion (RDF) crystallization: they create a porous diffusion network that suppresses convective flow and prevents the sedimentation of growing crystals. This ensures that nucleation occurs at the same location as crystal growth, maintaining the reaction-diffusion conditions necessary for fractal formation. As in gelatin, the shifts in fractal morphology (**Zones 1–3**) in agar can be explained by decreasing supersaturation and diffusion rate as the reaction front propagates outward. Moreover, the progressive increase in crystal size and distinct morphological transitions align with established literature on the role of supersaturation and diffusion rate in reaction-diffusion precipitation.

Agar contains agaropectin and pyruvate impurities, introducing ionizable functional groups into the gel matrix. This means that the pH of the medium can influence agar’s interaction with diffusing ions similar to gelatin, affecting both the ion diffusion coefficient and local supersaturation gradients.^52,53^ Additionally, agar’s pore size depends on ionic strength, which dynamically changes as supersaturation evolves.^27^ To assess the impact of ionic strength on zone formation, an experiment was conducted where NaCl (4.0 M) was added to the inner benzoate solution ([BZ] = 0.1 M) before allowing HCl to diffuse from the outer solution. As shown in Figure S7, this suppressed the formation of **Zones 2 and 3**, leaving only **Zone 1** intact. This result confirms that ionic strength plays a significant role in controlling the formation of distinct reaction-diffusion zones in agar, likely by modulating diffusion properties and supersaturation profiles.

The fundamental morphological difference between the BA fractals formed in agar and gelatin suggests a distinct crystallization mechanism in each system. One key observation is the difference in fractal color: in gelatin, BA fractals appear white, whereas in agar, they are transparent. This suggests differences in crystallite size, defect density, or light-scattering properties between the two systems. Additionally, while the crystal width in gelatin correlates with the pore size of the gelatin network, the crystal width in agar does not correlate with its average pore size (545 ± 4 nm),^27^ implying a different mode of crystal aggregation and growth regulation. Several studies have demonstrated that when crystals grow in gelatin, they tend to form white dendritic structures, whereas agar and agarose gels promote well-defined, compact crystal morphologies.^54–56^ This difference in crystallization behavior remains poorly understood, as the precise role of gelatin in promoting dendritic fractal growth is still an open question. One possibility is the difference in the charge of gelatin and agar: while agar has a negative charge (from agaropectin and pyruvates), gelatin has a positive charge at acidic pH, which is the pH range of our system (pH = 2.0–5.5). The difference in microcrystal structure to macro-fractal structure can be attributed to this difference in the charge of the diffusion medium. Further studies are needed to explore how gelatin-specific interactions influence nucleation dynamics and branching mechanisms, providing deeper insight into why diffusion-limited fractal growth manifests differently in different gel environments.

## Conclusions

This study presents a comprehensive investigation into the fractal growth mechanisms of benzoic acid (BA) crystallization within a reaction-diffusion framework (RDF), bridging experimental observations with diffusion-limited aggregation (DLA) theory and Monte Carlo simulations. By systematically varying gel networks (gelatin vs agar) and reactant concentrations, we demonstrated that fractal morphology is governed by diffusion constraints, supersaturation gradients, and gel-matrix interactions, establishing a new paradigm for fractal growth in soft-matter crystallization.

In gelatin-based systems, BA fractals exhibited dendritic growth with fractal dimensions converging toward classical diffusion-limited aggregation (DLA) values (∼1.71 to 1.74) at high supersaturation. A distinct three-zone transition emerged: **Zone 1** featured densely branched dendritic structures consistent with high nucleation rates, **Zone 2** exhibited branch thickening and reduced connectivity due to decreasing supersaturation and nucleation rate, and **Zone 3** showed dense, surface-attached crystallization, highlighting a shift away from fractal growth. Across all growth zones, uniform tensile macrostrain was observed, likely arising from the kinetically driven lattice distortions embedded during rapid, constrained growth in a gel medium that exerts mechanical resistance. The interplay between fractal assembly, diffusion constraints, and matrix confinement suggests that macrostrain is an inherent feature of reaction-diffusion-controlled crystallization rather than a localized anomaly.

The reverse-phase system, where sodium benzoate diffused inward instead of HCl, provided additional insight into diffusion-rate effects on fractal morphology. In this setup, branch thickening and a reduction in fractal dimension were observed, mirroring the transition from **Zone 1** to **Zone 2** in the normal-phase system. These findings strongly support the hypothesis that slower diffusion promotes compact growth by increasing the effective sticking coefficient and reducing fractal branching.

In agar-based systems, BA crystallization produced distinctly different morphologies compared to gelatin, forming spherulitic rather than dendritic fractals. Although a similar three-zone structure was observed, the morphological evolution differed markedly: **Zone 1** exhibited stacked rectangular plates, **Zone 2** developed elongated dendritic structures, and **Zone 3** presented irregular thread-like aggregates. Additionally, the role of ionic strength in zone formation was experimentally demonstrated by showing that increasing the NaCl concentration suppressed the formation of **Zones 2 and 3**. This observation highlights the significant impact of ionic strength on gel-network interactions, reinforcing the importance of electrostatic interactions between diffusing species and the agar gel matrix in modulating fractal morphogenesis.

By integrating Monte Carlo simulations, we established a quantitative framework linking supersaturation, diffusion, and fractal dimension. The remarkable agreement between experimental fractal dimension trends and simulated DLA structures suggests that reaction-diffusion crystallization follows universal stochastic aggregation principles, challenging the conventional view of molecular crystallization as purely deterministic.

This work lays the foundation for predictive control over fractal growth in reaction-diffusion systems, offering new strategies for hierarchical material design, biomimetic crystallization, and microstructured surface engineering. Expanding this framework to other organic acids, metal–organic frameworks (MOFs), and bioinspired materials could determine whether diffusion-controlled fractal growth is a universal phenomenon in self-assembled systems. Further studies into gel-matrix interactions, ionic strength effects, and pH-dependent structural modifications will be crucial for refining reaction-diffusion-based crystallization control.

Finally, this study opens avenues for applications in functional coatings, energy storage, nanostructured optics, and microfluidic self-assembly by demonstrating that reaction-diffusion crystallization can be harnessed to produce fractal architectures with tunable morphologies. The ability to engineer fractal growth through controlled reaction-diffusion conditions presents a powerful new approach for designing hierarchical materials with tailored properties, merging fundamental insights into stochastic growth with practical advancements in soft-matter physics and materials science.

## Supplementary Material





## Notes

Experimental procedure for 1D system, BA fractal without outer reactor, BA fractals in reverse-phase, and BA fractal in an agar-based system with 4.0 M NaCl (PDF)
Progression of the diffusion front visualized using bromocresol green (MOV)

## Abbreviations

BA: benzoic acid
BZ: benzoate
DLA: diffusion limited aggregation
RDF: reaction-diffusion framework
SEM: scanning electron microscope
PXRD: powder X-ray diffraction
