# Supplementary material for: Tunable Fractal Morphogenesis in Reaction-Diffusion Crystallization: From Dendrites to Compact Aggregates
Source: ACS Omega. 2025 Aug 5;10(34):38629–39. doi: 10.1021/acsomega.5c03338 (PMC12409535; doi:10.1021/acsomega.5c03338)
Supplement: Supplementary file 1 [file ao5c03338_si_001.pdf]

Tunable Fractal Morphogenesis in Reaction-Diffusion Crystallization: From Dendrites to Compact Aggregates

*Seungju Moon and Mazen Al-Ghoul**

Department of Chemistry, American University of Beirut, Beirut, Lebanon

Supporting Information

Experimental Procedure for 1D gelatin-based system

The inner gel phase was prepared by dissolving gelatin (8% w/w) in an aqueous sodium benzoate solution or diluted HCl solution at the desired concentration. The mixture was heated with continuous stirring until homogenized. The temperature was maintained below 45°C to prevent denaturation of gelatin. The resulting solution (6 mL) was poured into a glass tube with a diameter of 1.2 cm and a height of 12.0 cm. After one day, 1 mL of outer HCl solution (1.00 M) or outer sodium benzoate (1.00 M) was added above the gel.

Table S1. Values of full width at half maximum(°) of main peaks in the XRD pattern of the agar-based system and **Zones 1, 2, and 3** in the gelatin-based system.

	Zone 1	Zone 2	Zone 3	Agar
(002)	0.243	0.225	0.248	0.163
$(10\bar{1})$	0.241	0.220	0.245	0.166
(014)	0.250	0.223	0.252	0.198

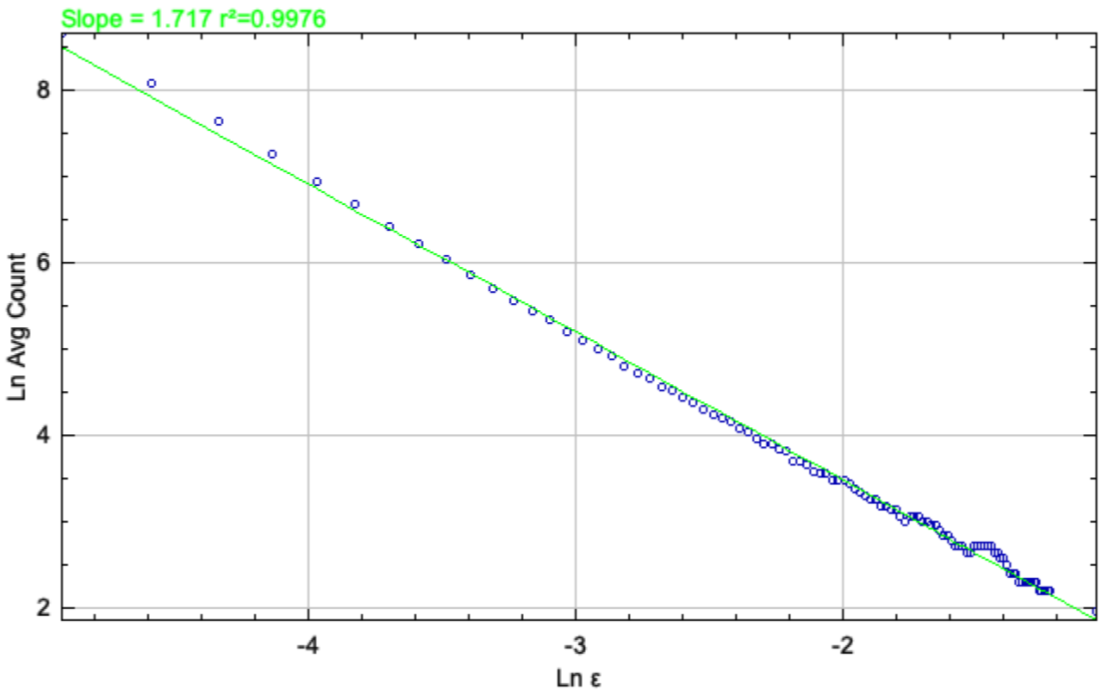

Figure S1. Plot of ln(average box count) versus ln(ε) using FracLac Box counting Plugin, where average box count represents the number of occupied boxes of size ε (ln N(ε) vs. ln (ε)). The negative slope of the plot yields the fractal dimension *D*.

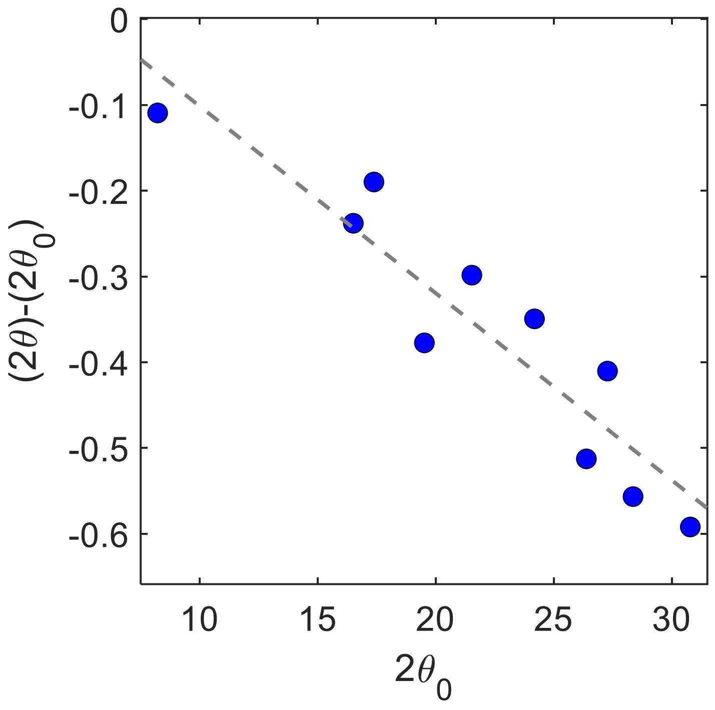

Figure S2. Plot of (2θ)-(2 θ_0) versus 2θ_0, where 2θ corresponds to the experimental PXRD peak positions of benzoic acid (BA) crystals from Zones 1, 2, and 3 in the gelatin-based system, and 2θ_0 represents the corresponding peak positions in the simulated BA pattern. The negative deviation that increases with 2θ0 is consistent with uniform tensile macrostrain, resulting in a systematic expansion of the lattice spacing across all zones. This behavior supports the interpretation that mechanical constraints imposed during gel-confined and surface-attached crystal growth introduce persistent lattice distortion across the entire system.

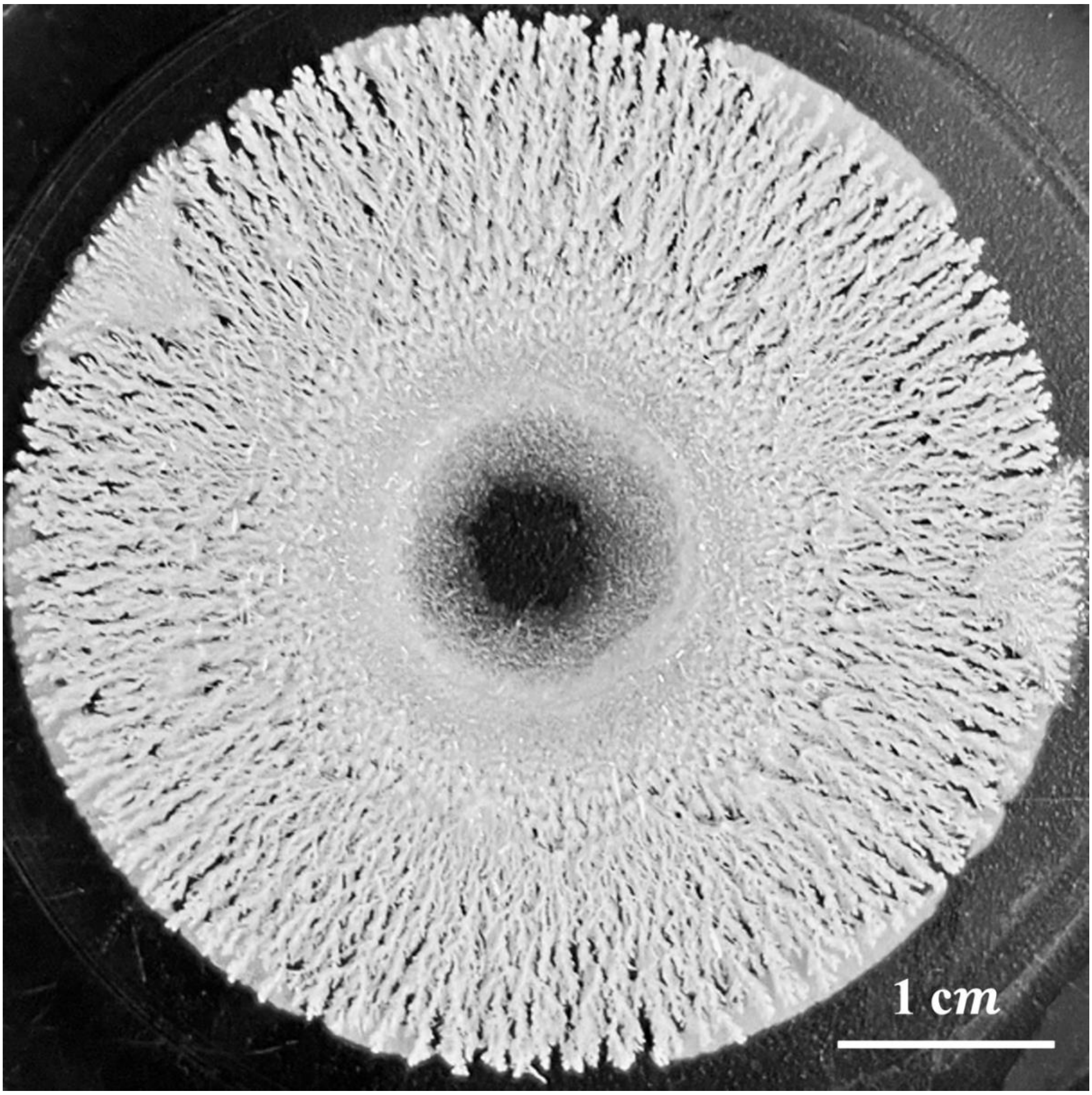

Figure S3. Optical image illustrating the BA fractal structure formed after 2 days of diffusion in a gelatin-based system without the outer reactor wall. The gel thickness was set at 0.2 mm, with an inner [BZ] of 0.10 M. This setup reveals a distinct bifurcation pattern emerging at the gel-air interface, highlighting intrinsic morphological behaviors driven by surface effects and emphasizing the inherent role of gelatin in mediating reaction-diffusion crystallization dynamics.

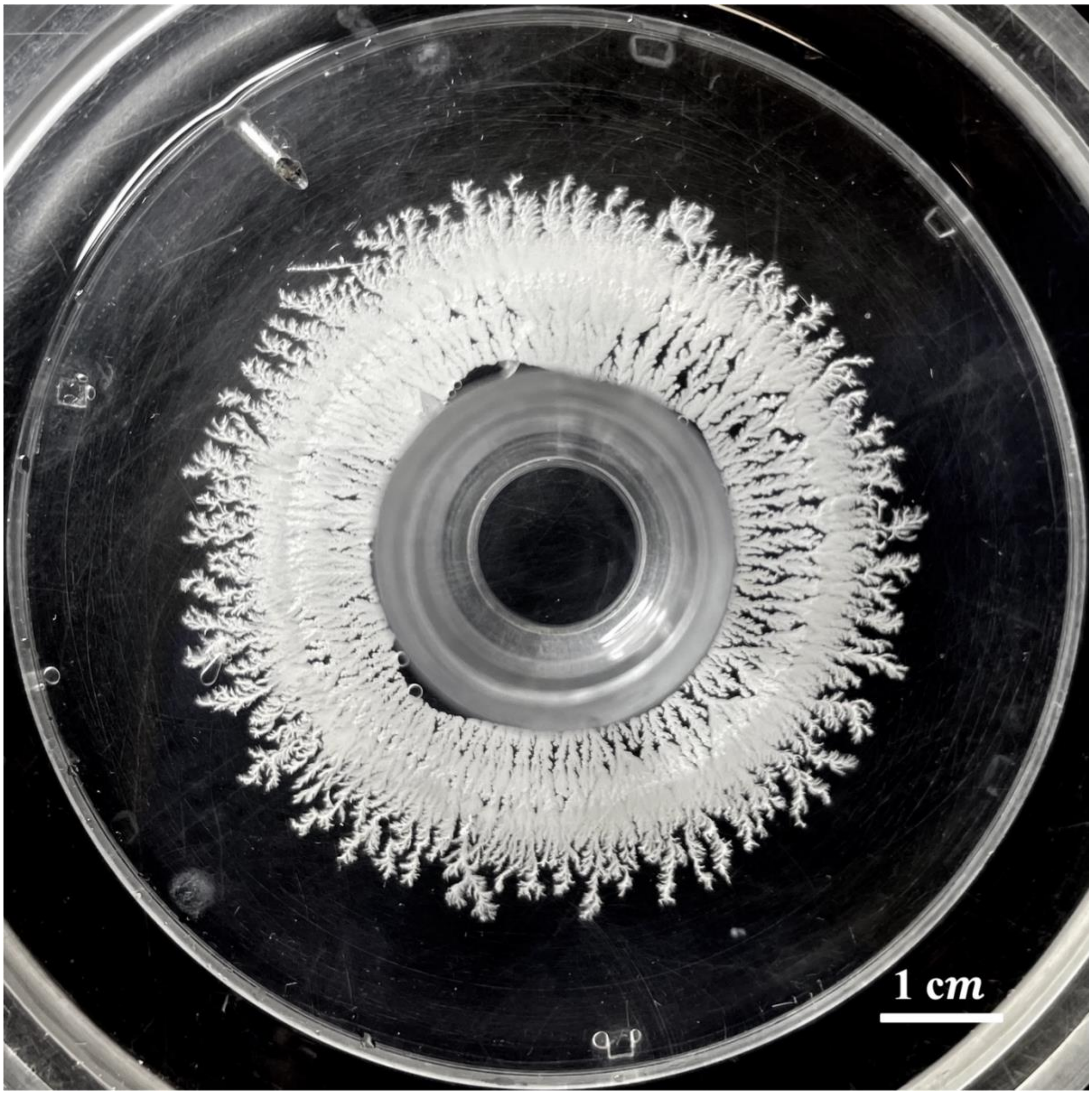

Figure S4. Optical image showing BA fractals formed after 2 weeks of diffusion in a 2D reactor under reverse-phase conditions (inner [H^+] = 0.10 M, outer [BZ] = 1.0 M) within a gelatin-based reaction-diffusion system. The prolonged diffusion time, resulting from slower benzoate ion diffusion compared to protons, leads to thicker dendritic branches and reduced fractal dimensions, highlighting the significant influence of diffusion dynamics on fractal morphogenesis.

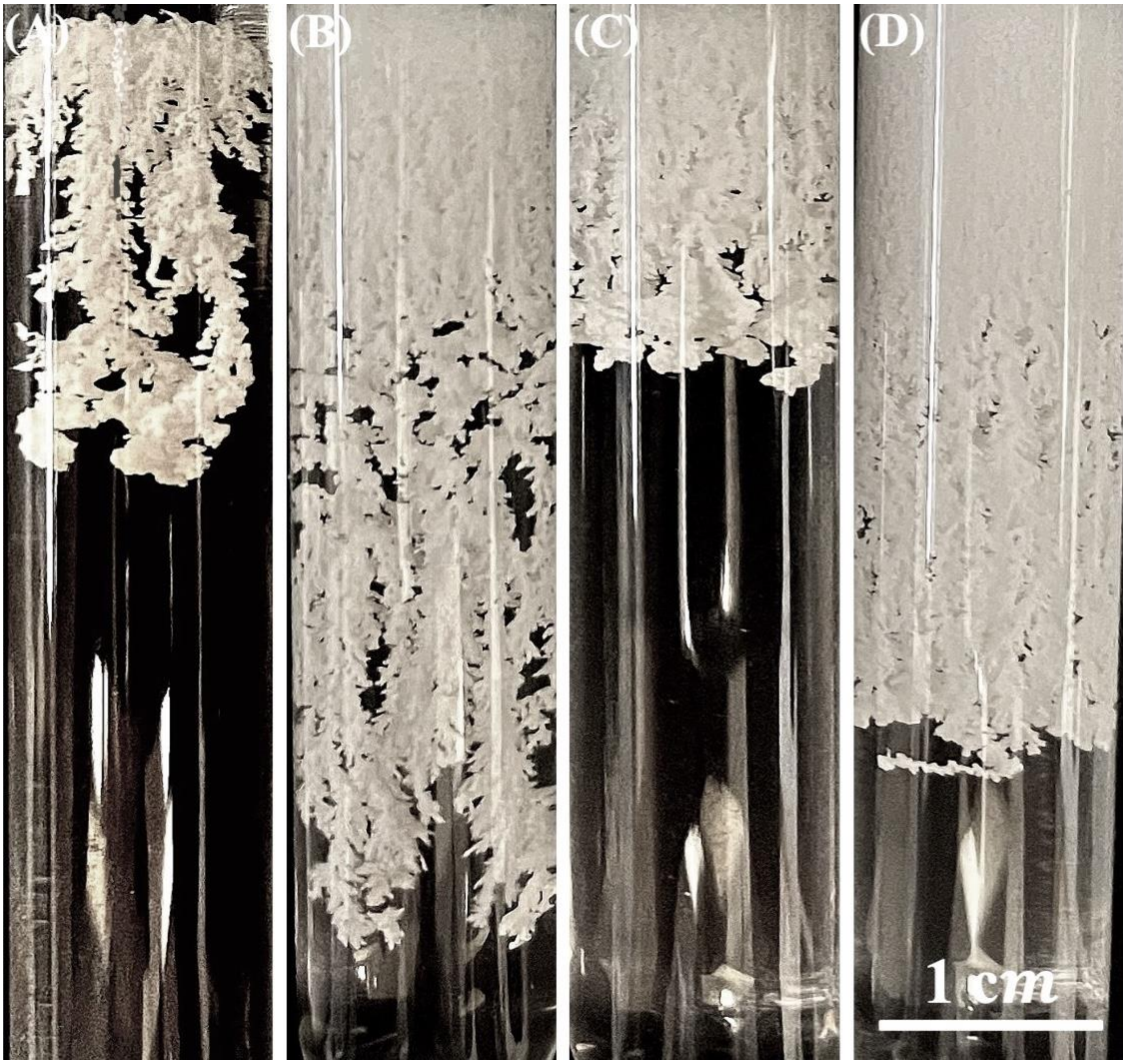

Figure S5. Optical images of BA fractals formed in 1D tubes under reverse-phase and typical-phase gelatin-based reaction-diffusion conditions. Conditions: (A) Reverse phase with inner [H^+] = 0.10 M and outer [BZ] = 1.0 M; (B) Typical phase with inner [BZ] = 0.10 M and outer [H^+] = 1.0 M; (C) Reverse phase with inner [H^+] = 0.15 M and outer [BZ] = 1.0 M; (D) Typical-phase with inner [BZ] = 0.15 M and outer [H^+] = 1.0 M. Images were captured after 2 weeks of diffusion for reverse-phase experiments and after 3 days for typical-phase experiments, highlighting the substantial effect of diffusion rate differences on fractal morphology and growth dynamics.

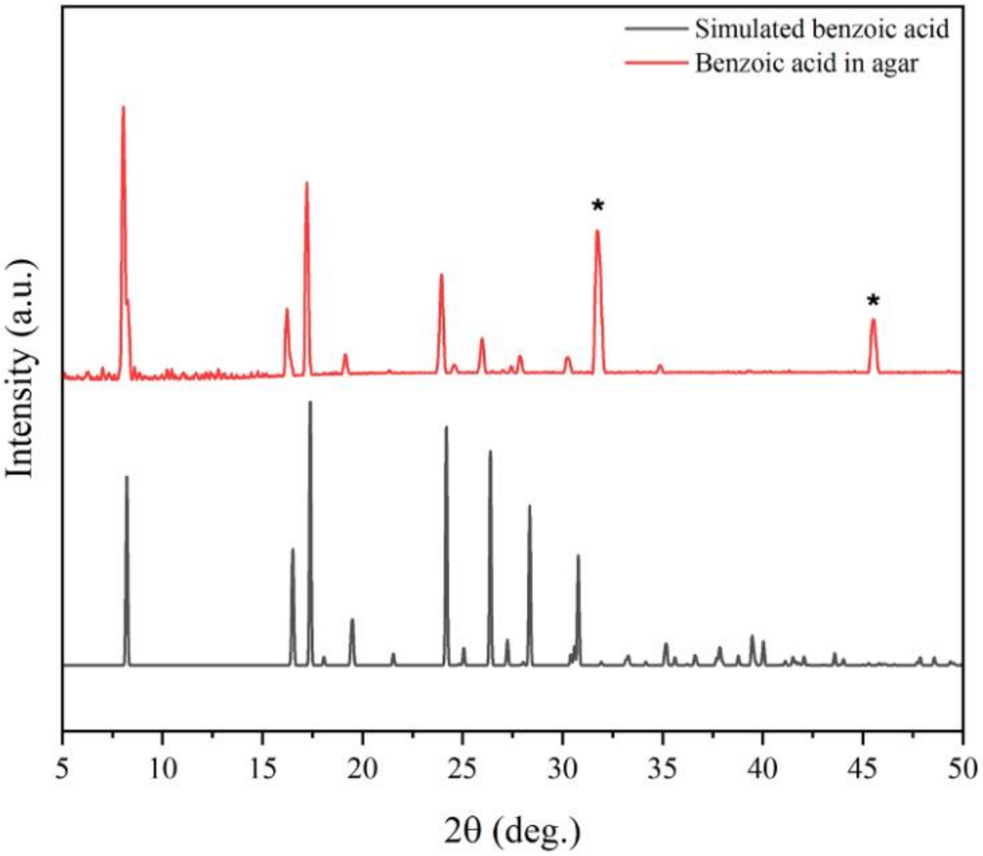

Figure S6. PXRD pattern of BA crystals obtained from the agar-based reaction-diffusion system. The diffraction peaks match the simulated benzoic acid crystal pattern, confirming phase purity and crystallinity. Peaks labeled with an asterisk (*) indicate minor NaCl impurities originating from the experimental conditions involving elevated ionic strength.

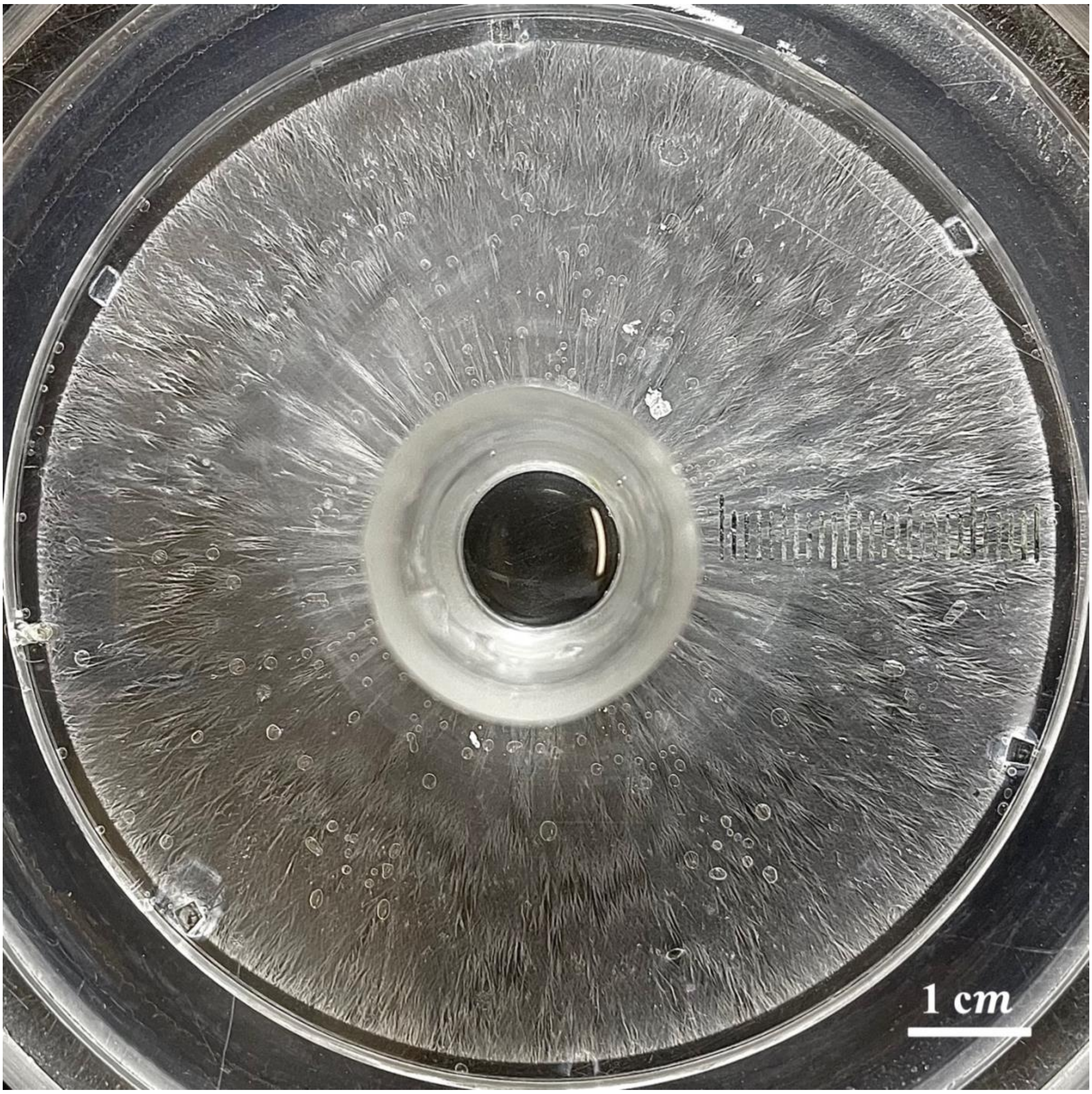

Figure S7. Optical image illustrating the fractal morphology of BA crystals formed in an agar-based reaction-diffusion system at inner [BZ] = 0.10 M and high ionic strength conditions (inner [NaCl] = 4.0 M). Under these conditions, only **Zone 1** is clearly formed, whereas **Zone 2 and Zone 3** are suppressed. This observation demonstrates the significant influence of ionic strength on morphological transitions, highlighting how elevated electrolyte concentration modifies the diffusion dynamics and gel-matrix interactions, consequently restricting fractal evolution.
